# Evaluation of the diagnostic role of radial probe endobronchial ultrasound for peripheral pulmonary lesions

**DOI:** 10.1111/crj.13792

**Published:** 2024-07-28

**Authors:** Wei Wang, Li Li, Qian Zhang, Yan Wang, Chun Hua Xu

**Affiliations:** ^1^ Department of Respiratory Medicine, Affiliated Nanjing Brain Hospital Nanjing Medical University Nanjing Jiangsu China; ^2^ Clinical Center of Nanjing Respiratory Diseases and Imaging Nanjing Jiangsu China; ^3^ Medical Imaging Department II, Affiliated Nanjing Brain Hospital Nanjing Medical University Nanjing Jiangsu China

**Keywords:** air bronchogram, continuous margin, diagnosis, heterogeneous, peripheral pulmonary lesions, radial probe endobronchial ultrasound

## Abstract

**Objective:**

To evaluate the predictive value of echo features of radial probe endobronchial ultrasound (RP‐EBUS) in the differential diagnosis of malignant and benign 1esions.

**Methods:**

The clinical data of 336 patients with peripheral pulmonary lesions (PPLs) undergoing RP‐EBUS were analyzed in order to evaluate the predictive value of the three EBUS echo features including continuous margin, absence of a linear‐discrete air bronchogram, and heterogeneous in pulmonary lesions. The sensitivity and specificity for each echoic feature or in combination in diagnosing malignancy or benignity were determined.

**Results:**

336 cases of PPLs including 216 cases of malignant lesions and 120 cases of benign lesions. The sensitivity and specificity of the continuous margin to the diagnosis of malignant lesions were 86.11% and 17.50%. The sensitivity and specificity of the absence of a linear‐discrete air bronchogram to the diagnosis of malignant lesions were 66.67% and 57.50%, and the positive predictive value was 73.85%. The sensitivity and specificity of heterogeneity to the diagnosis of malignant lesions were 65.28% and 72.50%, and the positive predictive value was 81.03%. The combination of heterogeneous and absence of a linear‐discrete air bronchogram could improve the diagnostic specificity to 87.50%, and the positive predictive value to 80.77%.

**Conclusion:**

The two EBUS echo features of heterogeneous and absence of a linear‐discrete air bronchogram have predictive value for PPLs, especially in the presence of two features the pulmonary lesions should be highly suspected malignant tumors.

## Graphical abstract

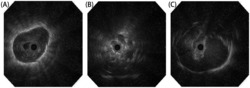

## 1 INTRODUCTION

With the development of imaging technology, chest multi‐slice spiral computed tomography (CT) has become a commonly used diagnostic method, and the detection rate of peripheral pulmonary lesions (PPLs) is gradually increasing. However, due to the complex etiology and lack of specific imaging manifestations, it is difficult to distinguish between benign and malignant lesions. In recent years, auxiliary technologies have been applied, including virtual bronchoscopy, magnetic navigation, radial ultrasound, and ultrafine bronchoscopy.^1^

The development of small‐caliber ultrasound probes has extended the clinical application of ultrasound examination to tracheal, bronchial, and PPLs. Under the guidance of fiberoptic bronchoscopy, small‐caliber ultrasound probes can successfully enter peripheral lesions. Endobronchial ultrasonography (EBUS) has been reported to help confirm the accurate insertion of bronchoscopy into the lesion site.^2^, ^3^ Under the guidance of EBUS, the diagnostic yield of peripheral lung cancer patients undergoing bronchoscopic lung biopsy significantly improved, while the incidence of complications did not increase.^4^ In addition, the use of guided sheath EBUS has been applied in the diagnosis of peripheral pulmonary lesions, which may increase the diagnostic yield of endoscopic biopsy in patients with fluoroscopically invisible pulmonary nodules.^2^ Recently, electromagnetic navigation bronchoscopy has attracted more attention due to its high diagnostic yield independent of lesion size and location, especially when lesions exceed the optical limitations of flexible bronchoscopy.^5^, ^6^

In addition to its role in improving the diagnostic yield of bronchoscopy biopsy for peripheral pulmonary nodules, the image features of EBUS also provide clues for determining the nature of peripheral abnormalities based on their different ultrasound features.^7^ A study has shown high consistency between EBUS images and histopathological results.^8^ EBUS has been reported to be very useful in determining the depth of tracheal and bronchial tumor infiltration by displaying the layered structure of the tracheal and bronchial walls. Previous reports have revealed that some characteristic echo patterns were associated with histopathological manifestations of benign and malignant lesions in gastrointestinal and lymph node colon cancer.^9^, ^10^ Based on the EBUS images of peripheral lung lesions, a previous report has correlated the histopathological results of surgical specimens to form an ultrasound image classification scheme to distinguish between benign and malignant lesions.^11^ At present, the application of EBUS echo features to predict the malignancy of lung lesions is emerging both domestically and internationally,^12^, ^13^, ^14^, ^15^ but research on PPLs is still very limited. This study evaluates the predictive value of radial probe endobronchial ultrasound (RP‐EBUS) echo characteristics for benign and malignant lesions by analyzing them.

## 2 MATERIALS AND METHODS

### 2.1 Patients

The subjects of this study were patients who underwent RP‐EBUS assessment of pulmonary lesions at Nanjing Chest Hospital from June 2020 to July 2022. During this period, a total of 1767 patients underwent bronchoscopy examinations with indications. Among 1767 patients, 516 patients with PPLs underwent RP‐EBUS assessment. Out of 516 patients, 473 showed lesions through RP‐EBUS. Among these patients, 137 were excluded from the study due to central lesions detected during bronchoscopy. The lesion position, boundary, size, vacuole sign, enhanced CT value, calcification, vascular cluster, speculation, lobulation, pleural indention bronchial, and lymph node change were recorded, and 336 patients with PPLs were finally enrolled. All patients underwent pathological diagnosis through bronchoscopy lung biopsy guided by EBUS. Patients whose biopsy results do not match clinical conditions will undergo CT‐guided percutaneous lung biopsy or surgical surgery. The biopsy results show non‐specific inflammatory lesions and will be followed up for at least 3 months after treatment. Exclusion criteria: Patients with abnormal coagulation function, severe cardiopulmonary dysfunction, a history of allergy to anesthetic drugs, and those with abnormal lesions in the tracheal lumen found during preoperative bronchoscopy.

The study was approved by the Ethics Committee of the Nanjing Brain Hospital, affiliated with the Nanjing Medical University. All patients expressed informed consent.

### 2.2 Equipment

The patient underwent flexible bronchoscopy (BF‐1 T260, Olympus, Japan) 5.8 mm in external diameter for complete inspection of airways before echo endoscopy. The EBUS (EU‐M30 S, Olympus, Japan) was integrated with a 20 MHz radial probe (UM‐S30–20 R, Olympus, Japan) 2.0 mm in external diameter and guide sheath kit (K‐203, Olympus, Japan).

### 2.3 Procedure

Preoperative preparation is the same as routine bronchoscopy examination. Fasting and drinking water are prohibited for 4–6 hours before the examination. During the operation, the patient is placed in a supine position, blood oxygen saturation and heart rate are monitored, nasal cannula is used for oxygen inhalation, venous access is established, and midazolam and fentanyl drugs are given for sedation and analgesia during the operation. After inserting the bronchoscope into the airway, a routine bronchoscopy examination is completed. Then, based on the chest CT, the position of peripheral pulmonary nodules is preliminarily determined. The ultrasound probe is sent through the bronchoscopy channel to the corresponding bronchus of the lesion, and the ultrasound scan is initiated. The surgeon slowly and uniformly enters and exits the ultrasound probe in the lesion area observes the ultrasound image and performs an EBUS examination on adjacent bronchioles to obtain the best ultrasound image. If the probe does not adhere well to the wall, The appearance of air interference artifacts can be achieved by injecting physiological saline through the bronchoscopy operation channel, and the resulting images are stored in the computer storage system.

### 2.4 Image analysis

**FIGURE 1:**
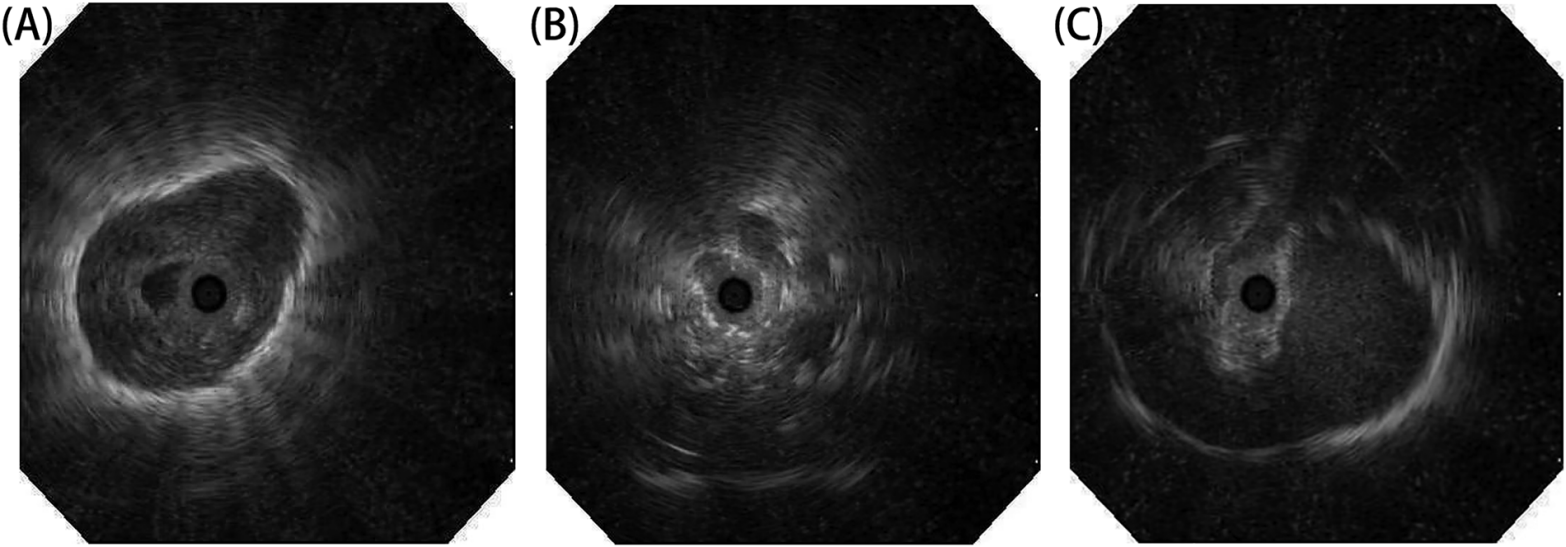
EBUS images. (A) Representative case of a continuous margin (B) linear‐discrete air bronchogram (C) heterogeneous echogenicity.

Analyze each EBUS image to determine if there were three types of echo features: continuous edges, air bronchogram, and heterogeneity. Analyze and predict the malignancy of pulmonary lesions based on these three types of echo characteristics. Continuous margin: Whether there was a continuous and clear margin line between the margin of the pulmonary lesions and adjacent lung tissue (Figure 1A). Air bronchogram: Whether point or short line hyperechoic signs were observed during the process of the ultrasound probe entering and exiting the lesion, this sign is the presence of air bronchogram within the lesion (Figure 1B). Heterogeneity: The internal structure of the lesion was characterized by a consistent intensity of EBUS echoes, which was considered homogeneous. For example, the intensity of EBUS echoes varies, indicating heterogeneity (Figure 1C). In order to determine whether these three features were present in the patient's EBUS images, two experienced bronchial ultrasound doctors independently judged all images and compared the results after reading the images. Among them, the continuous margin and the absence of linear‐discrete air bronchogram signs were consistent, while the heterogeneous sign was 5.75% inconsistent. Finally, consensus was reached through discussion.

### 2.5 Statistical analysis

All statistical analyses were performed using SPSS for windows version 22.0 (SPSS Inc., Chicago, IL, USA). The χ^2^ test was used to compare the difference of each variable in malignant or benign lesions. Odds ratios (ORs) and 95% confidence intervals (CIs) were adopted to assess the contribution of significant factors. Value P < 0.05 was considered statistically significant.

## 3 RESULTS

### 3.1 Hisotopathologic diagnosis of lesions

**Table** **1** summarizes the histopathological data of 336 cases of PPLs. Among these 336 patients, 216 were malignant lesions and 120 were benign lesions. Among malignant tumors, there were 174 cases (51.79%) of adenocarcinoma, 30 cases (8.93%) of squamous cell carcinoma, and 12 cases (3.57%) of small cell lung cancer. Among benign lesions, there were 48 cases of tuberculosis (14.29%), 15 cases of lung abscess (4.46%), 21 cases of pulmonary aspergillosis (6.25%), 15 cases of pneumonia (4.46%), 6 cases of pulmonary hamartoma (1.79%), and 15 cases of organized pneumonia (4.46%).

**TABLE 1:** Clinical characteristics of patients who underwent RP‐EBUS.

Characteristics	N
Patients	336
Sex	
Male	132 (39.29%)
Female	204 (60.71%)
Age years	58.7±10.2 (32‐76)
Lesion size (mm)	26.2±3.6 (8‐30)
Location of lesions on coronal CT	
1/3rd of central	186 (55.36%)
1/3rd of middle	102 (30.36%)
1/3rd of peripheral	48 (14.28%)
Lesion location	
Right upper lobe	69 (20.54%)
Right middle lobe	105 (31.25%)
Right lower lobe	84 (25.00%)
Left upper lobe	36 (10.71%)
Left lower lobe	42 (12.50%)
EBUS image	
Within	234 (69.64%)
Adjacent to	102 (30.36%)
Final diagnosis	
Malignant disease	216
Adenocarcinoma	174 (51.79%)
Squamous cell carcinoma	30 (8.93%)
Small cell lung cancer	12 (3.57%)
Benign disease	120
Tuberculosis	48 (14.29%)
Pneumonia	15 (4.46%)
Pulmonary aspergillosis	21 (6.25%)
Lung abscess	15 (4.46%)
Organizing pneumonia	15 (4.46%)
Pulmonary hamartoma	6 (1.79%)

RP‐EBUS, radial probe endobronchial ultrasound.

### 3.2 The characteristic patterns of EBUS images

Among 336 EBUS images of PPLs, 186 (86.11%) malignant lesions and 99 (82.50%) benign lesions had continuous margins, with no statistically significant difference (OR = 1.315, 95% CI: 0.716–2.417, P > 0.05). One hundred forty‐four cases (66.67%) of malignant lesions and fifty‐one cases (42.50%) of benign lesions absence of a linear‐discrete air bronchogram, and the difference was statistically significant (OR = 2.706, 95% CI: 1.709–4.284, P < 0.01). One hundred forty‐one cases (65.28%) of malignant lesions and thirty‐three cases (27.50%) of benign lesions showed heterogeneous in the internal structure, and the difference was statistically significant (OR = 4.956, 95% CI: 3.040–8.081, P < 0.01). The absence of a linear‐discrete air bronchogram and heterogeneity has a high predictive value for the benign and malignant pulmonary lesions, while the continuous margin has a low predictive value for the benign and malignant pulmonary lesions (**Table** **2**).

**TABLE 2:** Predictor of malignancy by RP‐EBUS image features.

Feature	Malignancy (n=216)	Benign (n=120)	OR	95% CI P	Value
Continuous margin	186 (86.11)	99 (82.50)	1.315	0.716‐2.417	0.428
Absence of linear discrete air bronchogram	144 (66.67)	51 (42.50)	2.706	1.709‐4.284	0.001
Heterogenicity	141 (65.28)	33 (27.50)	4.956	3.040‐8.081	0.001

### 3.3 Predictive values of malignant lesions

From a single EBUS echo feature perspective, the sensitivity of continuous margin in predicting malignant lesions was 86.11%, but the specificity was only 17.50%. The sensitivity and specificity of the absence of a linear‐discrete air bronchogram in predicting malignant lesions were 66.67% and 57.50%, respectively, with a positive predictive value of 73.85%. The sensitivity of heterogeneous echo prediction for malignant lesions was 65.28%, and the specificity was 72.50%, with a positive predictive value of 81.03%. The absence of a linear‐discrete air bronchogram combined with heterogeneous can improve diagnostic specificity (87.50%) and positive predictive value to 80.77%, but the sensitivity was only 29.17% (**Table** **3**).

**TABLE 3:** Prediction values of malignant features.

Features	Accuracy (%)	Sensitivity (%)	Specificity (%)	PPV (%)	NPV (%)	
A	alone	61.61	86.11	17.50	65.26	41.18
B	alone	63.39	66.67	57.50	73.85	66.67
C	alone	67.86	65.28	72.50	81.03	53.70
A and B	59.82	62.50	55.00	71.43	44.90	
A and C	52.68	36.11	82.50	78.79	41.77	
B and C	50.00	29.17	87.50	80.77	40.70	
A and B and C	50.89	27.78	92.50	86.96	41.57	

A=continuous margin; B=absence of linear discrete air bronchogram; C= heterogenicity; PPV, positive predictive value; NPV, negative predictive value.

**FIGURE 2:**
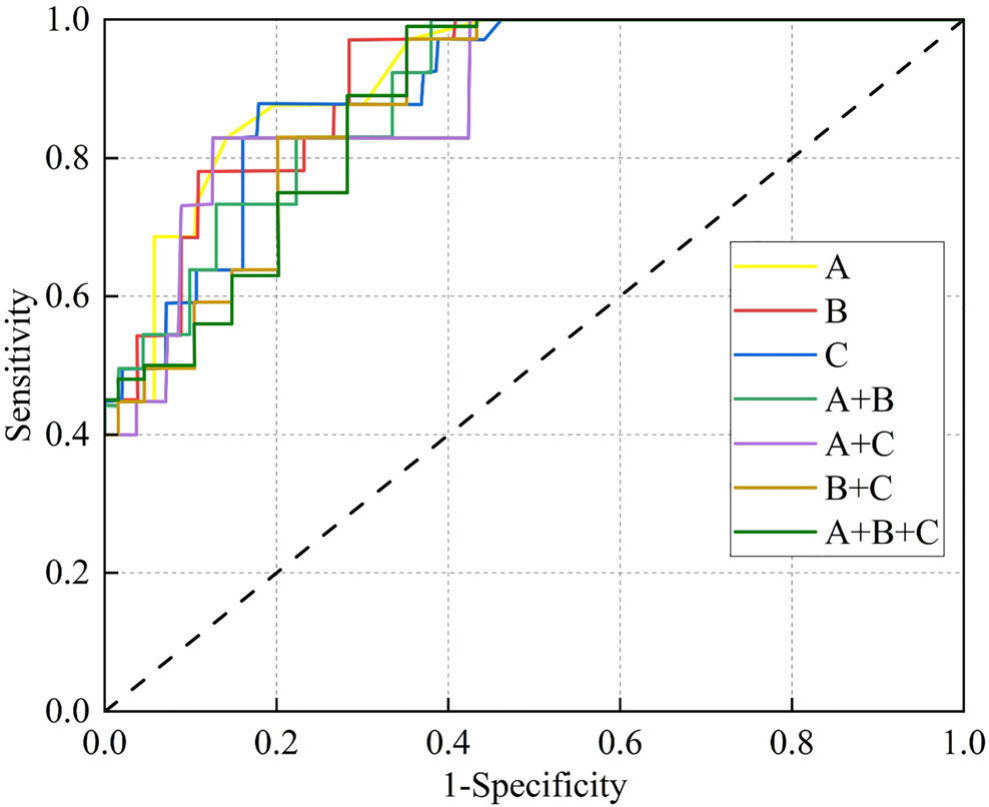
ROC curve analyses of the use of A, B, C, A and B, A and C, B and C, A and B and C to differentiate malignant and benign lesions.

The sensitivity of EBUS features absence of a linear‐discrete air bronchogram in predicting malignant lesions was higher in diameter < 10 mm than in diameter ≥ 10 mm (80.95% vs. 60.78%), but the specificity was lower (45.45% and 72.22%, respectively) (**Table** **4**). When the EBUS probe was adjacent to the lesion, the sensitivity of continuous margin prediction of EBUS features in malignant lesions was 55.56%, and when the EBUS probe was located within the lesion, it was 96.30% (**Table** **5**). Among the three echo features, A alone displayed the highest AUC and satisfactorily discriminated malignant lesions from benign lesions (Figure 2).

**TABLE 4:** Prediction of malignancy by RP‐EBUS feature with different tumor sizes.

Variables	Tumor size < 10 mm (n= 132)		10mm ≤ Tumor size ≤ 30 mm (n= 204)	
	Sensitivity (%)	Specificity (%)	Sensitivity (%)	Specificity (%)
Continuous margin	77.78	19.23	88.89	14.29
Absence of linear discrete air bronchogram	80.95	45.45	60.78	72.22
Heterogenicity	52.63	94.74	69.81	52.38

**TABLE 5:** Prediction of malignancy by RP‐EBUS features with different probe positions.

Variables	Probe within lesion (n= 234)		Probe adjacent to lesion (n= 102)	
	Sensitivity (%)	Specificity (%)	Sensitivity (%)	Specificity (%)
Continuous margin	96.30	25.00	55.56	6.25
Absence of linear discrete air bronchogram	80.77	69.23	30.00	35.71
Heterogenicity	69.09	82.61	52.94	58.82

## 4 DISCUSSION

How to diagnose PPLs quickly and accurately has always been a clinical difficulty.^16^ Clarifying whether it is a benign or malignant lesion often relies on bronchoscopic lung biopsy, CT‐guided puncture biopsy, and even thoracoscopic surgery. Although chest CT has a certain role in distinguishing between benign and malignant lesions, such as lobulated signs, spiciness signs, and pleural indentation signs in lung nodules suggesting the possibility of malignant nodules,^17^ it is still difficult to distinguish between benign and malignant properties for small pulmonary lesions. EBUS is an emerging respiratory intervention technology that can scan the interior or adjacent positions of peripheral lung lesions through bronchoscopy, which can clearly display the lesions and their fine internal structures, providing a new method and approach for the diagnosis of peripheral lung lesions.^18^ Previous studies had shown that the echo characteristics of EBUS were closely related to the pathological changes of the lesion tissue.^11^ Kurimoto et al were the first to establish a classification system for bronchial ultrasound image features of the internal structure of lesions, as a diagnostic tool for distinguishing between benign and malignant lesions.^11^ Previous studies have shown the value of various EBUS image features in distinguishing between benign and malignant, including lesion edge features, vascular signs, etc.,^14^, ^19^ but research on PPLs is relatively rare. This study selected three common and easily recognizable EBUS echo features to evaluate their predictive value for benign and malignant lesions.

Malignant tumors generally exhibit rapid and expansive growth, with the tumor body rapidly expanding and squeezing surrounding normal lung tissue, forming a thin layer of the lung atrophy zone, which is one of the reasons for the clear boundary of malignant tumors.^11^ Malignant tumors, due to invasive growth, generally contain very little or even no gas inside the tissue. Compared with lung tissue with a large amount of gas in the surrounding area, there is a significant difference in acoustic impedance between the two, and continuous margins are displayed in EBUS images.^14^ Generally, inflammatory lesions involving the lung interstitium and alveolar walls are mostly exudate and inflammatory cell aggregation. During the process of inflammatory expansion, the impact on surrounding lung tissue is inconsistent, and it is easy to appear as discontinuous margins in EBUS images.^20^ However, most benign lesions, such as tuberculosis, aspergilloma, and so on, are mostly localized proliferative lesions with little or even no exudation, so they appear as continuous margins on imaging. The results of this study indicated that the value of continuous margins in determining the malignant pulmonary lesions was not high. Many studies, both domestically and internationally, suggest that continuous margins in EBUS images may indicate the possibility of malignant tumors,^14^, ^20^ which may be related to different selected research subjects.

Air bronchogram refers to the presence of residual gas in the bronchi or alveoli of the lesion. During ultrasound scanning, the gas produces strong reflections and appears as a point or short line with high echogenicity on the ultrasound image. It is often located around the lesion and sometimes appears as a nearly concentric circular distribution. Bronchogenic carcinoma shows invasive growth, with cancer tissue often filling the affected bronchi and alveoli. Bronchial signs are relatively rare, but tumor invasion to the surrounding area also requires a process. A few bronchi may not be fully filled or have distal obstructive inflammation, which can also manifest as bronchial air signs. Inflammatory lesions often maintain the original shape of the bronchi, making it easier to form bronchial air signs.^11^ The results of this study showed that the absence of linear discrete air bronchogram in PPLs had a sensitivity positive predictive value of 86.11% and 65.26% for predicting benign and malignant lesions, respectively, but the specificity was not high, at 17.50%, lower than other scholars' research.^20^ This may be due to the small size of the pulmonary lesion and the limited involvement of the bronchioles, so air bronchogram was relatively rare in benign or malignant pulmonary lesions.

The growth process of malignant tumors is destructive, with the disappearance of normal lung tissue within the lesion replaced by a disorderly and irregular arrangement of tumor cells. The proportion of tumor cells and interstitial tissue in various parts of the lesion is often inconsistent, mixed with necrosis, bleeding, and interstitial fibrosis, resulting in heterogeneity of the structure of the tumor lesion on EBUS images.^11^ Inflammatory lesions generally maintain normal lung tissue structure, at least filling interstitial spaces or alveoli with inflammatory cells or substances, so benign lesions often exhibit homogeneity on EBUS images.^11^ This study also indicates that heterogeneity helps to distinguish between benign and malignant pulmonary lesions. The sensitivity and specificity of heterogeneity in predicting malignant tumors were 65.28% and 72.50%, respectively, which were lower than the research results of Kuo et al^14^ The reason may be that malignant nodules were still in the early stages of the disease, and there were generally fewer phenomena such as necrosis, bleeding, and interstitial fibrosis. This study showed that heterogeneity combined with the absence of linear discrete air bronchograms can improve the specificity of diagnosing malignant lesions, and the positive predictive value can also be increased to 80.77%.

The results of this study showed that although EBUS image features cannot replace biopsy, they had certain diagnostic values for predicting benign and malignant pulmonary lesions. The absence of linear discrete air bronchogram and heterogeneity of the lesion suggest the possibility of malignant lesions, especially when both features exist simultaneously, high suspicion of malignant tumors is necessary.

## AUTHOR CONTRIBUTIONS

Wei Wang, Yan Wang, and ChunHua Xu designed the study. Li Li and Qian Zhang collected data. Chunhua Xu and Yan Wang wrote the paper.

## CONFLICT OF INTEREST STATEMENT

The authors declare no competing interests.

## ETHICS STATEMENT

The study was approved by the Ethics Committee of the Affiliated Nanjing Brain Hospital, Nanjing Medical University. All patients expressed informed consent.

## Data Availability

The data that support the findings of this study are available from the corresponding author upon reasonable request.
